# Gut microbiota-dependent metabolite trimethylamine *N*-oxide (TMAO) and cardiovascular risk in patients with suspected functionally relevant coronary artery disease (fCAD)

**DOI:** 10.1007/s00392-022-01992-6

**Published:** 2022-02-26

**Authors:** Melissa Amrein, Xinmin S. Li, Joan Walter, Zeneng Wang, Tobias Zimmermann, Ivo Strebel, Ursina Honegger, Kathrin Leu, Ibrahim Schäfer, Raphael Twerenbold, Christian Puelacher, Noemi Glarner, Thomas Nestelberger, Luca Koechlin, Benjamin Ceresa, Philip Haaf, Adam Bakula, Michael Zellweger, Stanley L. Hazen, Christian Mueller

**Affiliations:** 1 Department of Cardiology, Cardiovascular Research Institute Basel (CRIB, University Hospital Basel, University of Basel, Petersgraben 4, CH-4031 Basel, Switzerland; 2 Department of Cardiovascular and Metabolic Sciences, Lerner Research Institute, Cleveland Clinic, 9500 Euclid Avenue, Cleveland, OH 44195 USA; 3 Department of Radiology, University Hospital Zürich, University of Zürich, Zürich, Switzerland; 4 Department of Internal Medicine, University Hospital Basel, University of Basel, Basel, Switzerland; 5 Departement of Cardiology, University of British Columbia, Vancouver, Canada; 6 Department of Cardiac Surgery, University Hospital Basel, University of Basel, Basel, Switzerland; 7 Department of Cardiovascular Medicine, Heart and Vascular Institute, Cleveland Clinic, 9500 Euclid Avenue, Cleveland, OH 44195 USA

**Keywords:** Trimethylamine *N*-oxide (TMAO), Incident major adverse cardiac events (mace), Cardiovascular death, Myocardial infarction, Functionally relevant coronary artery disease (fCAD), Gut microbiota

## Abstract

**Background:**

Trimethylamine N-oxide (TMAO) has been associated with cardiovascular outcomes. However, the diagnostic value of TMAO and its precursors have not been assessed for functionally relevant coronary artery disease (fCAD) and its prognostic potential in this setting needs to be evaluated.

**Methods:**

Among 1726 patients with suspected fCAD serum TMAO, and its precursors betaine, choline and carnitine, were quantified using liquid chromatography tandem mass spectrometry. Diagnosis of fCAD was performed by myocardial perfusion single photon emission tomography (MPI-SPECT) and coronary angiography blinded to marker concentrations. Incident all-cause death, cardiovascular death (CVD) and myocardial infarction (MI) were assessed during 5-years follow-up.

**Results:**

Concentrations of TMAO, betaine, choline and carnitine were significantly higher in patients with fCAD versus those without (TMAO 5.33 μM vs 4.66 μM, *p* < 0.001); however, diagnostic accuracy was low (TMAO area under the receiver operating curve [AUC]: 0.56, 95% CI [0.53–0.59], *p* < 0.001). In prognostic analyses, TMAO, choline and carnitine above the median were associated with significantly (*p* < 0.001 for all) higher cumulative events for death and CVD during 5-years follow-up. TMAO remained a significant predictor for death and CVD even in full models adjusted for renal function (HR = 1.58 (1.16, 2.14), *p* = 0.003; HR = 1.66 [1.07, 2.59], *p* = 0.025). Prognostic discriminative accuracy for TMAO was good and robust for death and CVD (2-years AUC for CVD 0.73, 95% CI [0.65–0.80]).

**Conclusion:**

TMAO and its precursors, betaine, choline and carnitine were significantly associated with fCAD, but with limited diagnostic value. TMAO was a strong predictor for incident death and CVD in patients with suspected fCAD.

**Clinical trial registration:**

NCT01838148.

**Graphical abstract:**

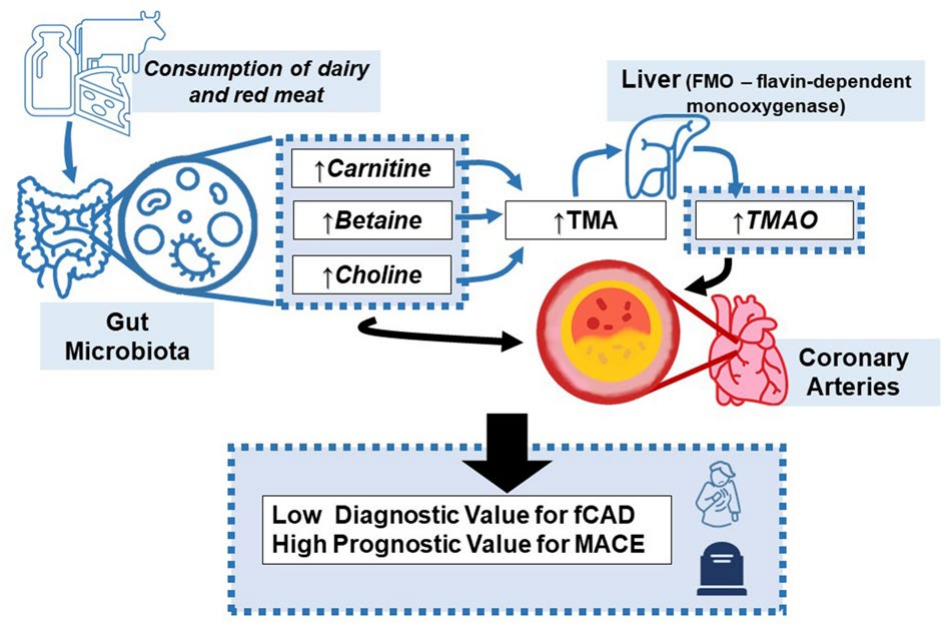

**Supplementary Information:**

The online version contains supplementary material available at 10.1007/s00392-022-01992-6.

## Introduction

During the last decade, translational research has highlighted intestinal microbiota as possible mediators between dietary habits and both the development and progression of coronary artery disease (CAD) [1–5]. The consumption of red-meat and dairy products rich in choline, betaine, carnitine, and trimethyllysine leads to the production of trimethylamine (TMA) by certain intestinal microbiota [1, 6, 7]. In a second step, TMA is oxidized to trimethylamine N-oxide (TMAO) by flavin monooxygenase in the liver [1, 8–12]. TMAO seems to induce systemic inflammation at least in part by the activation of the NF-κB pathway and the increased expression of pro-inflammatory cytokines including TNF-α and IL-1β [6, 13–16]. TMAO was indicated in multiple studies to accelerate atherosclerosis and enhance platelet reactivity as well as thrombosis potential [3, 17–19]. In addition, recent studies have documented an association between plasma TMAO concentration and the risk of death, myocardial infarction (MI), and stroke in patients with either stable CAD or acute coronary syndromes [1, 2, 4, 10, 20–22].

Beyond possible therapeutic opportunities, this insight suggests that TMAO and/or its precursors might have prognostic and/or diagnostic utility in the non-invasive detection of CAD, particularly the more aggressive CAD phenotype leading to myocardial ischemia during everyday activities (functionally relevant CAD, fCAD). Even more importantly, TMAO may provide prognostic utility in identifying those at incident risk for hard clinical endpoints including death and MI. For this indication, TMAO and/or its precursors may help physicians in the selection of patients for cardiac work-up including invasive or non-invasive coronary imaging [23, 24]. Given the high number of patients with very low pre-test probability for fCAD referred for sophisticated cardiac imaging including myocardial perfusion scanning, and with cardiac imaging causing annual costs of more than $500 million in the United States alone, biomarker guidance may have substantial medical and economic value [23, 25]. Therefore, the aim of this study was to prospectively assess the clinical prognostic utility performance and the diagnostic accuracy of circulating TMAO and its precursors on all-cause death, cardiovascular death, AMI and the composite endpoint of CV death and AMI, in patients with suspected fCAD.

## Methods

### Study design and oversight

This analysis is part of a large ongoing prospective diagnostic study (NCT01838148, clinicaltrials.gov) designed to advance the early detection of fCAD [26, 27]. The local ethics committee approved the study, which was carried out according to the principles of the Declaration of Helsinki. All patients provided written informed consent. The authors designed the study, gathered, analyzed and vouched for the data and analysis, wrote the paper, and made the decision to submit it for publication. Reported data follow STARD guidelines for studies of diagnostic accuracy [28].

### Patient population

Patients were recruited from 2010 to 2016 at the University Hospital of Basel, Switzerland. Enrolled patients were suspected to have fCAD and were referred for rest/stress myocardial perfusion single-photon emission tomography/computer tomography (MPI-SPECT). MPI-SPECT/CT was the preferred cardiac imaging technique in patients with a wide range of pre-test probabilities for fCAD during that time. Patients requiring chronic dialysis were excluded.

### Quantified clinical assessment

The likelihood for the presence of fCAD was quantified by the integrated clinical judgment of the treating cardiologist using a visual analogue scale (VAS) ranging from 0 to 100% twice: once before stress testing integrating all medical information available at that time, such as age, sex, cardiovascular risk factors, previous cardiac history, symptoms and baseline ECG data; second, after stress testing, integrating symptoms the patient experienced during exercise/stress, the workload achieved, and ECG changes recorded during exercise/stress. The cardiologist was blinded to both biomarker measurements and MPI-SPECT images at the time of assessment.

### Blood sampling and laboratory methods

Venous non-fasting blood samples for determination of TMAO, choline, betaine, carnitine, and high-sensitivity cardiac troponin (hs-cTn) T [23, 26, 29], an established cardiovascular biomarker associated with fCAD, were obtained at rest, before stress testing. After centrifugation, samples were frozen at − 80 °C until assayed in a blinded fashion in a dedicated core laboratory. Serum TMAO, choline, betaine and carnitine were quantified using stable isotope dilution LC/MS/MS analyses as previously described using a Shimadzu Nexera Ultra High Performance Liquid Chromatograph (UHPLC) system interfaced with Shimadzu 8050 Triple Quadrupole Mass Spectrometer [1, 3, 18]. Hs-cTnT was measured with the Elecsys System on the Modular Analytics E170 or the Cobas e 602 (Roche Diagnostics, Rotkreuz, Switzerland). Limit of blank and limit of detection of this assay are 3.0 ng/L and 5.0 ng/L, respectively. The upper reference limit (URL) of a healthy reference population was 14 ng/L with an imprecision corresponding to 10% coefficient of variation (CV) at 13 ng/L [29]. Creatinine was measured by either the University Hospital of Basel central laboratory, Risch laboratories and Rothen laboratories. eGFR (estimated glomerular filtration rate) was calculated using the CKD-EPI (Chronic Kidney Disease Epidemiology Collaboration) equation provided within the *transplantr*-package in R. Cystatin-C was measured by SomaLogic using the SomaScan^®^ assay, which uses single-stranded DNA-based protein affinity reagents that are modified to mimic amino acid chains, enhancing protein-nucleic acid interaction [30]. These so-called SOMAmer reagents are selected against proteins in their native folded state, which after binding and other processing steps can then be quantified by DNA quantification techniques, providing a readout in relative fluorescent units (RFU) directly proportional to the amount of the protein [30].

### Adjudication of the presence of fCAD

Expert interpretation of MPI-SPECT/CT images combined with information obtained from invasive coronary angiography and whenever available fractional flow reserve measurements were used in the adjudication of fCAD.

All patients underwent a routine rest/stress dual isotope (201Tl for rest, 99mTc sestamibi for stress) or single isotope (99mTc sestamibi for stress and rest) MPI-SPECT protocol as described previously [27, 31, 32]. MPI-SPECT images were scored semi-quantitatively using a 17-segment model with a 5-point scale (0 = normal, 1 = mildly reduced tracer uptake, 2 = moderately reduced tracer uptake, 3 = severely reduced tracer uptake and 4 = no uptake). The summed rest score (SRS) and summed stress score (SSS) were calculated based on the 17 segments in the rest and stress images. The difference of SRS and SSS yielded the summed difference score (SDS), whereby an SDS of at least two or a positive transient ischemic dilation ratio (TID) was considered as inducible myocardial ischemia. Two readers derived SSS and SRS by visual assessment and compared with the software (QGS) result. Differences in the visual assessment by the two readers were resolved by consensus. In case of equivocal findings from MPI-SPECT and coronary angiography, two independent cardiologists (one interventional cardiologist, one general cardiologist) that were blinded to biomarker results reviewed the case. A positive perfusion scan was overruled when coronary angiography showed normal coronary arteries and a negative perfusion scan was overruled if coronary angiography (within three months) revealed a high-grade coronary lesion (> 75% or fractional flow reserve (FFR) lower than 0.80) [23, 26, 29].

### Adjudication of major adverse cardiac events

The prognostic endpoints were all-cause death, cardiovascular death and MI during long-term follow-up. Patients were contacted by telephone or standardized follow-up letter 1 year, 2 years and 5 years after enrolment with ongoing follow-up. In case of an event, further information was obtained from hospital records, general practitioner/cardiologists records, or the national death registry blinded to biomarker concentrations.

### Statistical analysis

Normality testing was done using visual assessments (histograms and QQ-plots) and as Shapiro–Wilk test. Continuous variables are presented as median and respective interquartile range (IQR) and categorical variables are presented as frequencies and respective percentages. The Agresti-Coull method was used to calculate confidence intervals of proportions. Measures of central tendency of the biomarkers were compared by Mann–Whitney *U* test. Baseline characteristics were compared by Kruskal test for continuous data and Fisher test for categorical data. Diagnostic accuracy of the clinical assessment, TMAO, betaine, choline, carnitine, and their combination for fCAD was quantified by the area under the receiver operating curve (AUC) and compared with the method described by DeLong et al. [33]. Based on previous findings showing an association between TMAO concentration and prevalent CAD [6, 11, 34], subgroup analysis was performed stratified according to the presence or absence of previously known CAD. We hypothesized that TMAO concentration would have highest diagnostic accuracy in patients without known CAD. Using logistic regression with fCAD as outcome, log2-transformed concentrations of TMAO or one of its precursors (betaine, choline, carnitine) were adjusted for patient characteristics, risk-factors and treatment, and combined with the quantified clinical assessment of the treating physician before and after stress testing. Multicollinearity was checked by the variance inflation factor (VIF) using the vif-function of the car package in R-Studio.

Kaplan–Meier curves for concentrations of TMAO, betaine, choline and carnitine below and above the median as well as stratified by the upper reference limit of the established biomarker hs-cTnT (URL 14 ng/L), were constructed and compared by log rank testing. Quantification of the predictive accuracy of TMAO and precursors was done by time dependent AUCs whilst accounting for censoring (timeROC package) [35]. Cox regression analysis was used to evaluate whether the analyzed biomarkers were independent predictors of patient characteristics and risk factors. Assumptions for Cox regression were tested using Schoenfeld residuals. Statistical analyses were performed with R version 4.0.2. All hypothesis testing was two-tailed, and a *p* value < 0.05 was considered statistically significant.

## Results

### Characteristics of patients

Overall, 1726 consecutive patients with suspected functionally relevant coronary artery disease (fCAD) were included in this analysis (Supplemental Fig. 1), 478 patients (28%) were adjudicated to have fCAD. A total of 421 (24%) patients underwent coronary angiography with 284 patients within the 3 months after enrolment. Anamnesis revealed 764 (44%) to have known history of CAD. During follow-up, 88 patients experienced an incident MI, 223 patients died overall, 115 of which died due to cardiovascular reasons (CV death). Characteristics of patients stratified by fCAD are shown in Table 1. Stratification of patients by experience of either an AMI, all-cause or CV death, is shown in Supplemental Table 1. Patients with fCAD tended to be older and a higher proportion were male. A significant proportion of patients had diabetes, hypertension, and a history of cardiovascular disease. To portrait renal state of the patients, baseline eGFR and cystatin-C were added to the baseline tables. As expected, eGFR was significantly lower in patients with fCAD and cystatin-C significantly higher in patients with fCAD.

**Table 1:** Patient baseline characteristics

	Overall	fCAD		*p* value
	*N* = 1726	No (*N* = 1248)	Yes (*N* = 478)	
Age (years; median [IQR])	69.0 [61.0, 77.0]	68.0 [59.0, 76.0]	71.0 [63.0, 78.0]	< 0.001
Sex (Male (%))	1133 (65.6)	752 (60.3)	381 (79.7)	< 0.001
BMI (median [IQR])	27.3 [24.4, 30.9]	27.1 [24.2, 30.4]	27.7 [24.8, 31.6]	0.017
Medical history				
Diabetes (%)	447 (25.9)	266 (21.3)	181 (37.9)	< 0.001
Ever smoker (%)	1070 (62.0)	750 (60.1)	320 (66.9)	0.009
Family history of CAD (%)	514 (29.8)	371 (29.7)	143 (29.9)	0.953
History of hypertension (%)	1375 (79.7)	965 (77.3)	410 (85.8)	< 0.001
History hypercholesterolemia (%)	1229 (71.2)	858 (68.8)	371 (77.6)	< 0.001
History of CAD (%)	764 (44.3)	494 (39.6)	270 (56.5)	< 0.001
History of MI (%)	450 (26.1)	270 (21.6)	180 (37.7)	< 0.001
History of PCI (%)	592 (34.3)	384 (30.8)	208 (43.5)	< 0.001
History of bypass (%)	236 (13.7)	139 (11.1)	97 (20.3)	< 0.001
History of PAD (%)	166 ( 9.6)	99 (7.9)	67 (14.0)	< 0.001
History of heart failure (%)	54 ( 3.1)	30 (2.4)	24 (5.0)	0.008
Aortic valve disease (%)				< 0.001
None	1477 (85.6)	1092 (87.5)	385 (80.5)	
Stenosis	97 ( 5.6)	54 (4.3)	43 (9.0)	
Insufficiency	149 ( 8.6)	100 (8.0)	49 (10.3)	
Combined	3 ( 0.2)	2 (0.2)	1 (0.2)	
Mitral valve disease (%)				< 0.001
None	1336 (77.4)	1004 (80.5)	332 (69.5)	
Stenosis	2 ( 0.1)	2 ( 0.2)	0 ( 0.0)	
Insufficiency	387 (22.4)	241 (19.3)	146 (30.5)	
History of Stoke or TIA (%)	141 ( 8.2)	93 ( 7.5)	48 (10.0)	0.095
History of COPD (%)	159 ( 9.2)	115 ( 9.2)	44 ( 9.2)	1.000
Baseline medication				
Aspirin (%)	1015 (58.8)	687 (55.0)	328 (68.6)	< 0.001
Thienopyridine (%)	112 ( 6.5)	69 ( 5.5)	43 ( 9.0)	0.012
Nitroglycerine (%)	147 ( 8.5)	77 ( 6.2)	70 (14.6)	< 0.001
Beta-Blocker (%)	906 (52.5)	597 (47.8)	309 (64.6)	< 0.001
Calcium-Antagonist (%)	387 (22.4)	287 (23.0)	100 (20.9)	0.367
Amiadarone (%)	42 ( 2.4)	27 ( 2.2)	15 ( 3.1)	0.294
Diuretic (%)	706 (40.9)	483 (38.7)	223 (46.7)	0.003
ACE-Inhibitor (%)	519 (30.1)	342 (27.4)	177 (37.0)	< 0.001
AR-Blocker (%)	576 (33.4)	408 (32.7)	168 (35.1)	0.333
Statin (%)	993 (57.5)	671 (53.8)	322 (67.4)	< 0.001
Phemprocoumon (%)	201 (11.6)	121 ( 9.7)	80 (16.7)	< 0.001
Proton pump Inhibitor (%)	543 (31.5)	392 (31.4)	151 (31.6)	0.954
VAS before Ergo (median [IQR])	40 [20, 60]	30 [19, 49]	50 [30, 70]	< 0.001
VAS after Ergo (median [IQR])	40 [20, 60]	30 [19, 49	50 [30, 76]	< 0.001
Echo_LVEF (median [IQR])	58 [50, 62]	60.0 [54.0, 62.5]	55.0 [45.0, 60.0]	< 0.001
eGFR_baseline (median [IQR])	79.8 [60.0, 92.1]	82.0 [63.6, 93.7]	75.9 [55.2, 88.5]	< 0.001
Cystatin_C [RFU/1000]	2.5 [2.2, 3.0]	2.4 [2.1, 2.9]	2.6 [2.3, 3.3]	< 0.001
TMAO (median [IQR])	4.8 [3.2, 7.6]	4.7 [3.1, 7.2]	5.3 [3.5, 8.8]	< 0.001
Betaine (median [IQR])	34.6 [28.3, 43.1]	34.2 [27.8, 42.4]	35.8 [28.9, 44.6]	0.003
Choline (median [IQR])	14.4 [12.4, 16.9]	14.3 [12.3, 16.8]	14.8 [12.8, 17.1]	0.011
Carnitine (median [IQR])	39.4 [34.4, 44.9]	39.1 [34.1, 44.7]	40.3 [35.5, 46.2]	0.002

*ACE* [angiotensin-converting enzyme] inhibitor, *ARB* Angiotensin-II Receptor Blockers, *BMI* body mass index, *CABG* Coronary artery bypass grafting, *COPD* chronic obstructive pulmonary disease, *PAD* peripheral artery disease, *PCI* Percutaneous coronary intervention, *TIA* transient ischemic attack, *VAS* clinical assessment for presence of fCAD before/after ergometry but prior to imaging; Cystatin-C in relative fluorescent unit (RFU)/1000)

### Circulating TMAO, betaine, choline and carnitine concentrations

**Fig. 1:**
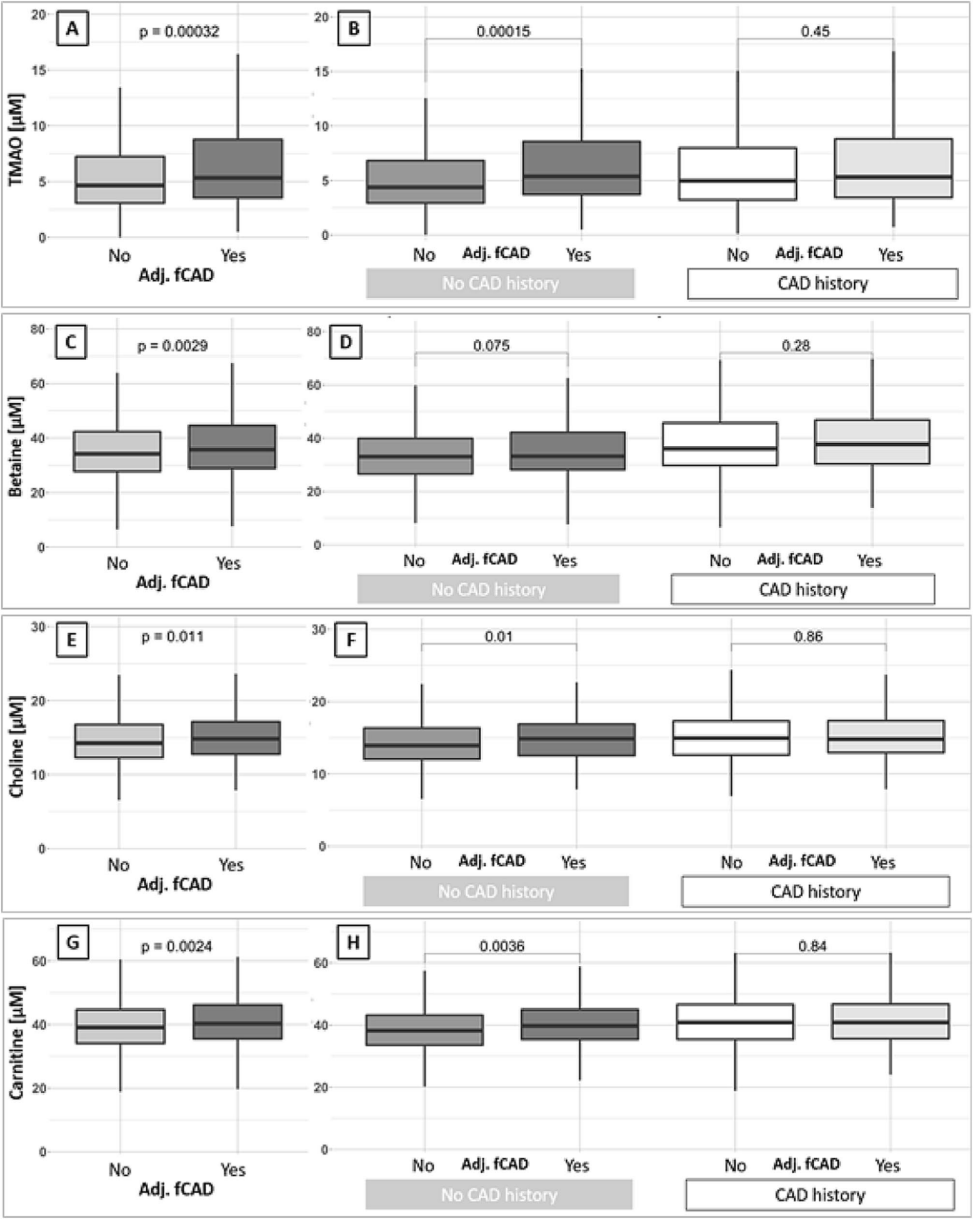
Levels of TMAO, betaine, choline and carnitine compared between patients adjudicated to have fCAD (**A**, **C**, **E**, **G**) and compared between patients adjudicated to have fCAD within subgroups of patients with and without history of CAD (**B**, **D**, **F**, **H**)

Median TMAO concentrations were significantly higher in patients with fCAD than in those without fCAD (5.33 IQR [3.55, 8.80] µM vs. 4.66 IQR [3.08, 7.24] µM, *p* < 0.001, Fig. 1A). Stratification by a history CAD revealed that this phenomenon was exclusively present in patients without known CAD (5.36 IQR [3.71, 8.57] µM vs 4.38 IQR [2.94, 6.83] µM, *p* < 0.001, Fig. 1B). Similar findings emerged for the three precursors betaine, choline and carnitine (Fig. 1C–H).

In patients with normal eGFR (*n* = 689), TMAO and the three precursors were significantly higher in patients adjudicated to have fCAD (Supplemental Fig. 2A–D). The baseline characteristics of this subgroup are presented in Supplemental Table 2.

TMAO concentrations were significantly, albeit weakly, correlated with hs-cTnT (Spearman’s rho 0.32, *p* < 0.001), NT-proBNP (Spearman’s rho 0.22, *p* < 0.001) and age (Spearman’s rho 0.25, *p* < 0.001). These correlations were weaker for betaine, choline and carnitine (Supplemental Fig. 3).

### Diagnostic performance for the detection of fCAD

In the overall cohort, diagnostic accuracy for detection of fCAD was quantified and showed modest but statistically significant value for TMAO with a ROC AUC of (0.56, 95% CI 0.53–0.59, *p* < 0.001) and its precursors (betaine 0.55, 95% CI 0.52–0.58, *p* = 0.002; choline 0.54, 95% CI 0.51–0.57, *p* = 0.007; carnitine: 0.55, 95% CI 0.51–0.58; *p* = 0.001; Supplemental Fig. 4). Similar findings emerged in the subgroup of patients without known CAD (TMAO 0.59, 95% CI [0.54–0.63], *p* < 0.001; betaine 0.54, 95%-CI [0.50–0.58], p = 0.039; choline: 0.56, 95% CI [0.51–0.60], *p* = 0.006; carnitine 0.57, 95% CI [0.52–0.61], *p* = 0.002; Supplemental Fig. 4B). TMAO and its precursors did not significantly increase the AUC provided by the quantitative clinical assessment by the treating physician before (AUC 0.61, 95% CI [0.58–0.64]) and after (AUC 0.65, 95% CI [0.62–0.68]) stress testing (*p* > 0.05 for all comparisons of VAS + TMAO (or precursors) versus VAS alone, Supplemental Fig. 4C + 4D). TMAO (OR 1.19, 95% CI [1.08–1.31], *p* < 0.001) and its precursors were significant predictors of fCAD in the univariable model, with TMAO remaining a significant predictor even after adjusting for age, sex and CAD history (OR 1.12, 95% CI [1.01–1.24], *p* = 0.036, Supplemental Table 3). When adjusting for renal function (i.e., taking Cystatin-C into the model) or when adjusting for further for pre-defined patient characteristics, cardiovascular risk factors and medical history (model 2), neither TMAO nor its precursors remained significant predictors. In patients with normal eGFR, TMAO and the three precursors were only significant predictors in the univariable model but were no longer significant after adjusting further (Supplemental Table 3).

### Prognostic performance for incident major adverse cardiac events

The median follow-up time was 1827.5 days (IQR [756, 1908]). The cumulative event incidence for 5-years death was 12.9% (223 events), for 5-years CV death 6.7% (115 events) and for 5-years MI 5.1% (88 events), and the composite 5-years MACE (MI and cardiovascular death) was 11.8% (203 events).

In Kaplan–Meier analyses, TMAO concentrations above the median had substantially higher all-cause, CV mortality and mace events versus patients below the median (*P* < 0.001, each; Supplemental Fig. 5 and 6). Similar results emerged for choline and carnitine.

**Fig. 2:**
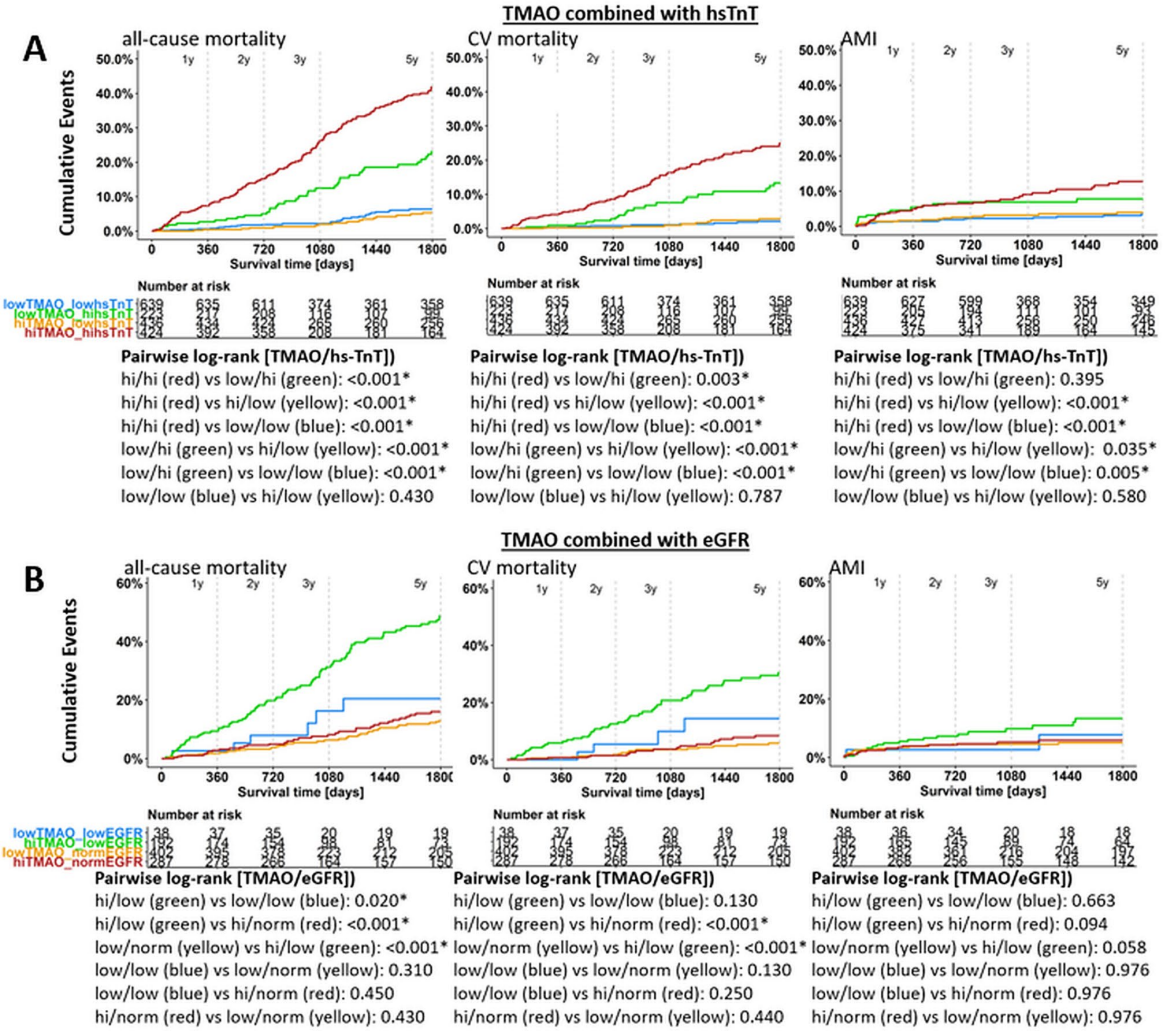
Kaplan Meier survival analysis of TMAO combined with low and high levels hs-Troponin (below/above URL of 14 ng/L; panel **A**) and TMAO combined with low and normal ranges of eGFR (> 60 mL/min/1.73m^2^ considered normal eGFR; panel **B**—in patients with available eGFR data (*n* = 921))

Kaplan–Meier analyses revealed that stratification according to hs-TnT and TMAO concentrations below/above the URL provided incremental prognostic value with the highest all-cause and CV mortality observed in patients with both hs-cTnT and TMAO above the URL (Fig. 2A).

Subgroup analysis (*n* = 919) of marker-combination with low and normal eGFR levels, Kaplan–Meier survival analyses revealed significantly highest cumulative all-cause death events in patients with low eGFR combined with a TMAO above the median (Fig. 2B).

**Fig. 3:**
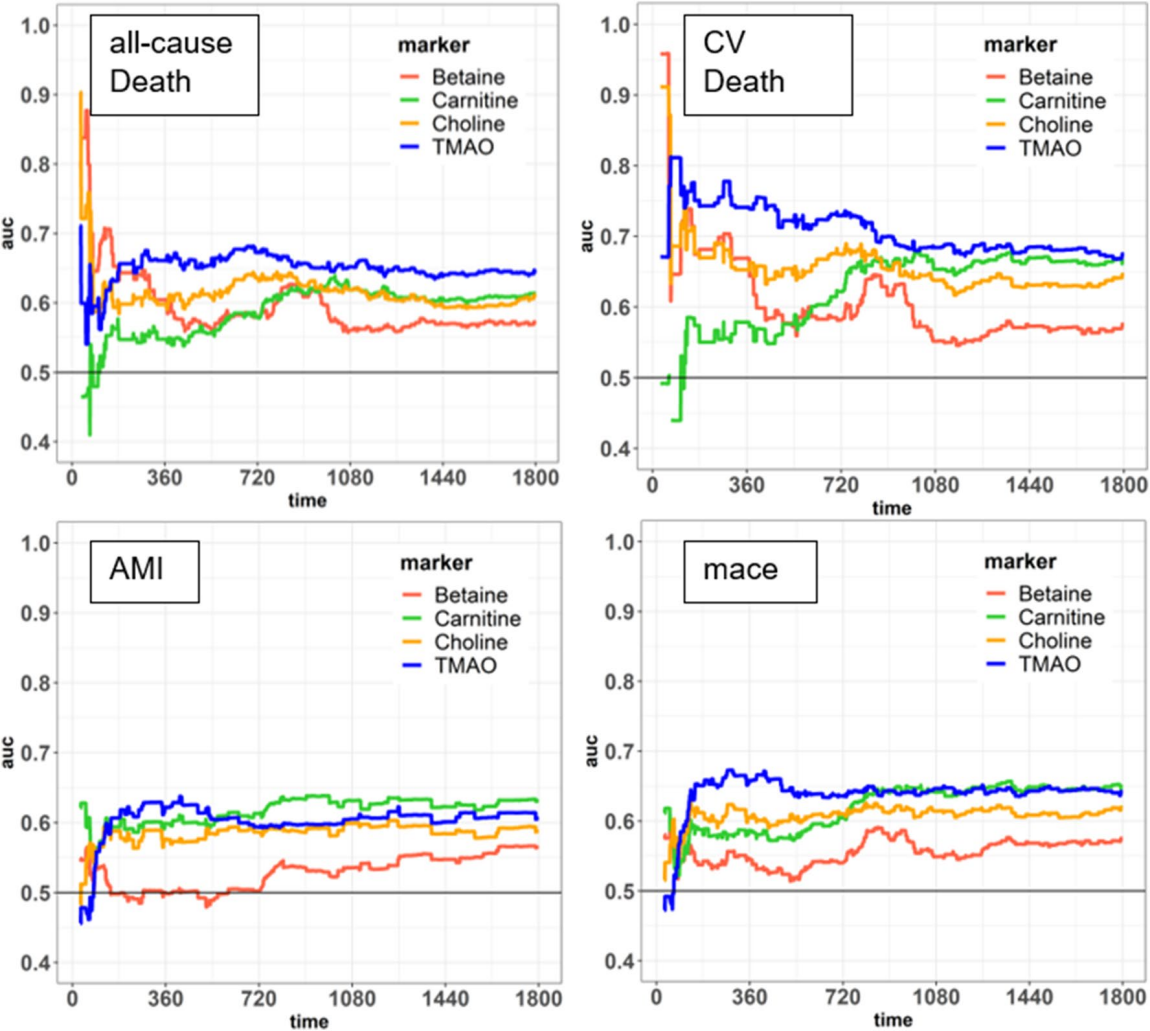
Time-dependent AUC of ROC of the four markers and over 5-years for all-cause death (223 events), cardiovascular death (115 events), AMI (88 events) and mace (203 events). Each step represents a change in AUC due to an event or censoring. AUC in the first days (ca. 180) vary due to small number of events in early stages

Time-dependent ROC curve analysis showed a consistent and moderate-to-good discriminative performance of TMAO and choline for 5-years all-cause death (e.g. TMAO at 2-years AUC: 0.67, 95% CI [0.61–0.73]; Choline at 2-years AUC: 0.64, 95%-CI [0.57–0.70]) and 5-years CV death (TMAO at 2-year, AUC: 0.73, 95% CI [0.65–0.81]; Choline at 2-year AUC: 0.67, 95% CI [0.59, 0.76]), and modest accuracy for the other markers and endpoints (Fig. 3). Furthermore, Cox-regression analyses on continuous log-transformed markers are shown in Table 2. TMAO, Carnitine and Choline remained independent predictors in the full model without adjustment of renal function (model 2). When adjusting further for renal function (model 2 + cystatin-c) both TMAO and carnitine remained independent predictors for 5-years CV death. In the cox-regression analysis performed on median stratified marker (Supplemental Table 4), only TMAO remained a significant predictor of all-cause death and CV death, even when adjusted fully and taking renal function into account (model 2 + cystatin-c).

**Table 2:** Hazard ratios of the univariate and adjusted Cox regression models with continuous log2-transformed markers for the outcomes all-cause death, cardiovascular death and acute myocardial infarction (CI—confidence interval; HR—Hazard Ratio)

	Outcome measure	Univariate HR (95% CI), *p* value	Model 1 HR (95% CI), *p* Value	Model 1 + cystatin-C	Model 2 HR (95% CI), *p* value	Model 2 + cystatin-C
TMAO	All-cause death	1.42* (1.29, 1.58), * p* < 0.001	1.28* (1.13, 1.43), * p* < 0.001	1.16* (1.03, 1.30, * p* = 0.013	1.23* (1.09, 1.38), * p* < 0.001	1.11 (0.99, 1.26), *p* = 0.084
	CV death	1.60* (1.39, 1.84), * p* < 0.001	1.44* (1.4, 1.68), * p* < 0.001	1.28* (1.10, 1.48), * p* < 0.001	1.36* (1.16, 1.58), * p* < 0.001	1.19* (1.01, 1.40), * p* = 0.032
	AMI	1.38* (1.17, 1.65), * p* < 0.001	1.33* (1.11, 1.60), * p* = 0.002	1.22* (1.01, 1.48), * p* = 0.043	1.32* (1.08, 1.60), * p* = 0.006	1.17 (0.95, 1.45), *p* = 0.146
Betaine	All-cause death	1.52* (1.16, 1.99), * p* = 0.002	1.27 (0.96, 1.68), * p* = 0.090			
	CV death	1.86* (1.28, 2.70), * p* = 0.001	1.57* (1.07, 2.32), * p* = 0.023	1.49* (1.00, 2.20), * p* = 0.048	1.62* (1.09, 2.41), * p* = 0.017	1.45 (0.98, 2.15) *p* = 0.061
	AMI	1.30 (0.86, 1.96), *p* = 0.219				
Choline	All-cause death	3.77* (2.59, 5.49), * p* < 0.001	2.46* (1.62, 3.73), * p* < 0.001	1.95* (1.31, 2.90), * p* < 0.001	2.10* (1.38, 3.21), * p* < 0.001	1.60* (1.06, 2.42), * p* = 0.026
	CV death	5.99* (3.62, 9.91), * p* < 0.001	4.29 (2.45, 7.49), * p* < 0.001	3.01* (1.79, 5.04), * p* < 0.001	3.53* (2.00, 6.24), * p* = 0.001	2.36* (1.34, 4.14), * p* = 0.003
	AMI	2.71* (1.49, 4.91), * p* = *0.001*	2.15* (1.08, 3.79), * p* = 0.017	1.53 (0.83, 2.84), * p* = 0.170	1.90* (1.01, 3.55), * p* = 0.046	1.30 (0.68, 2.49), *p* = 0.436
Carnitine	All-cause death	3.16* (2.11, 4.28), * p* < 0.001	2.24* (1.48, 3.37), * p* < 0.001	1.67* (1.12, 2.49), * p* = 0.012	2.00* (1.32, 3.04), * p* = 0.001	1.54* (1.02, 2.32), * p* = 0.041
	CV death	6.24* (3.68, 10.58), * p* < 0.001	4.45* (2.59, 7.67), * p* < 0.001	3.01* (1.78, 5.07), * p* < 0.001	3.60* (2.06, 6.26), * p* < 0.001	2.43*(1.41, 4.19), * p* = 0.001
	AMI	3.39* (1.84, 6.22),* p* < 0.001	2.58* (1.39, 4.78),* p* = 0.003	2.01* (1.09, 3.72), * p* = 0.025	2.79* (1.49, 5.25), * p* = 0.001	2.09* (1.09, 4.00), * p* = 0.026
Subset (*n* = 689): subset of patients with normal eGFR data						
TMAO	All-cause death	1.06 (0.76, 1.17), *p* = 0.570				
	CV death	1.13 (0.81, 1.56) *p* = 0.470				
	AMI	1.10 (0.80, 1.52), *p* = 0.561				

Model 1 adjustment: age, gender and history of coronary artery disease; Model 2 adjustment: pre-defined patient characteristics, cardiovascular risk factors and medical history including age, gender, body mass index, smoking history, positive cardiovascular family history, hypertension, hypercholesterolemia, history of diabetes, history of stroke/TIA, history of CAD, previous AMI, history of heart failure and adjudicated functionally relevant coronary artery disease

*p < 0.05

## Discussion

The accurate, non-invasive and inexpensive detection of fCAD is a major unmet clinical need. So too is the accurate and minimally invasive ability to identify those at increased risk for incident adverse events. The present study suggests that TMAO has clinical prognostic value for the identification of subjects at risk for incident adverse cardiac events, with modest, albeit significant, capacity to predict risk of fCAD. Recent translational insights suggest TMAO as a potential mediator of atherosclerosis [1, 2, 10, 36, 37]. Even more so, recent mechanistic studies suggest TMAO contributes to subject vulnerability for adverse events like thrombosis (MI and stroke) through both heightened platelet responsiveness, and enhanced artery wall inflammatory signaling [38, 39]. We, therefore, tested the hypothesis that TMAO, and its precursors betaine, choline and carnitine, may provide diagnostic and/or prognostic value in a large prospective study including 1726 patients with suspected fCAD referred for cardiac work-up using MPI-SPECT. We report five major findings.

*First*, TMAO, betaine, choline and carnitine concentrations were significantly higher in patients with fCAD versus those without fCAD. This is in line with prior pilot studies reporting that TMAO concentrations were positively associated with atherosclerotic burden in stable CAD patients [2, 40]. Interestingly, the difference observed was largely restricted to patients without known CAD history, the cohort in whom a diagnostic test is, arguably, more relevant. Also, TMAO was demonstrated to be associated with plaque instability [41]—therefore, TMAO possibly was only significantly elevated in that subgroup as the marker may play a role in soft plaques, rather than in calcified plaques. *Second*, TMAO and precursor marker levels were lower in patients with normal eGFR (> 60 mL/min per 1.73 m^2^) and patients adjudicated to have fCAD had significantly higher marker levels compared to those without. Notably, high TMAO in combination with low-eGFR were at significantly higher risk of all-cause death compared to low-TMAO combined with low-eGFR (*n* = 921). This is in line with previous findings demonstrating clearance of TMAO by the kidney and showing its association with reduced renal function [42–45]. Baseline eGFR additionally was significantly lower in patients with fCAD, supporting findings of lower eGFR being associated with increased risk of coronary artery disease [46–48]. Taking renal function, therefore, into account in the adjusted cox regression models, TMAO, choline and carnitine remained significant predictors for CV death. *Third*, while the AUC for fCAD for TMAO and its precursors was relatively low, it was statistically significant. Plasma levels of TMAO and its precursors (choline, betaine and carnitine) did not provide incremental diagnostic value for prediction of fCAD to clinical judgment of the cardiologist. Nonetheless, all markers were significant risk factors for fCAD in the univariate model, with TMAO remaining significant in models adjusted for age, sex and CAD history. *Fourth*, in contrast, concentrations of TMAO, carnitine and to a lesser extent choline were significant predictors of incident adverse events including either MI, CV-death, or all cause death. This finding is in concordance with previous reports where TMAO has been shown to be associated with all-cause and CV-death [1, 2, 4, 10, 20–22]. Moreover, significantly more adverse events (all-cause or CV death, or AMI) occurred in patients with high TMAO in combination with a hs-cTnT above the URL, a quantitative marker of cardiomyocyte injury strongly associated with future CV-death [23, 49]. *Fifth*, time-dependent ROC curve analysis showed consistent moderate-to-good prognostic accuracy of TMAO and its precursors for all-cause and CV-death during follow-up. The prognostic value for TMAO persisted also in fully adjusted models, highlighting possible implications for routine clinical practice given similar observations in recent studies [4, 20, 27, 36]. Overall, the prognostic accuracy for all-cause and CV-death was highest and most consistent for TMAO, and slightly lower and less consistent for its precursors and other outcomes. The same has been found in a stable CAD cohort where elevated TMAO likewise was associated with higher long-term mortality risk. [11]

Several therapeutic strategies could result in a reduction of TMAO concentrations: reduction in intake of red meat, rich in TMA precursors; use of targeted treatment of selected microbiota, e.g. by application of specific oral antibiotics to eradicate the intestinal microbiota responsible for TMA production; and inhibition of flavin monooxygenase in the liver [2, 3, 34, 50]. Clinical intervention studies could further elucidate whether TMAO is a true causal mediator or a bystander in atherothrombosis.

This study has important strengths, including central adjudication of fCAD, adjudication of hard outcomes, and prospective examination of the relationship of TMAO with respect to fCAD and incident MI, CVD death, and all-cause mortality over a long-term follow-up of 5 years. In addition, the study examines the relationship of multiple nutrient TMAO precursors betaine, choline and carnitine in a large prospective study of patients in need of active decision making. Also, several limitations should be considered when interpreting the findings of this study: first, these data were generated in a single-center study. While single-center studies are by definition prone to selection bias, generalizability of these findings seems high as standardized patient consenting, clinical work-up with MPI-SPECT/CT as the initial imaging modality was performed in patients with a wide range of pre-test probability for fCAD, and longer term outcome ascertainment was complete. Second, despite using a very stringent methodology for the adjudication of fCAD, we might still have misclassified a small number of patients, which would result in an underestimation of the true accuracy of TMAO and its precursors for fCAD. This is overcome by the examination of the relationship between TMAO and time dependent incident hard adverse events. Third, the cohort consisted of a predominantly Caucasian population, not allowing the analysis of other ethnicities due to their under-representation. Finally, we cannot comment on the dietary association of TMAO or precursor level, as this was not recorded in the frame of the study, nor the possible role of TMAO and its precursors in patients with terminal kidney failure on chronic dialysis, as these subjects were excluded.

In conclusion, TMAO and its precursors choline and carnitine showed potential prognostic value for short- and long-term risk-stratification for hard clinical events including CV-death, albeit with limited diagnostic utility in patients with suspected fCAD.

## Supplementary Information

Below is the link to the electronic supplementary material.

Supplementary file1 (DOCX 999 KB)

## Data Availability

Please contact the corresponding author regarding data availability.

## Code availability

Please contact the corresponding author to request codes.
