# Supplementary material for: Gut microbiota-dependent metabolite trimethylamine N-oxide (TMAO) and cardiovascular risk in patients with suspected functionally relevant coronary artery disease (fCAD)
Source: Clin Res Cardiol. 2022 Feb 26;111(6):692–704. doi: 10.1007/s00392-022-01992-6 (PMC9151506; doi:10.1007/s00392-022-01992-6)
Supplement: Supplementary file 1 — Supplementary file1 (DOCX 999 KB) [file 392_2022_1992_MOESM1_ESM.docx]

**SUPPLEMENT - Gut microbiota-dependent metabolite trimethylamine N-oxide and cardiovascular risk in patients with suspected functionally relevant coronary artery disease**

1757 consecutive patients with clinical suspicion of functionally relevant coronary artery disease and available TMAO, betaine, choline and carnitine concentrations enrolled between November 2013 and April 2015.

1726 patients available for analysis

- 25 patients with terminal renal failure requiring chronic haemodialysis

- 6 patients without available follow-up data

- 46 patients without available clinical assessment of the treating physician

**Supplemental Fig. 1** – Flowchart of the study population


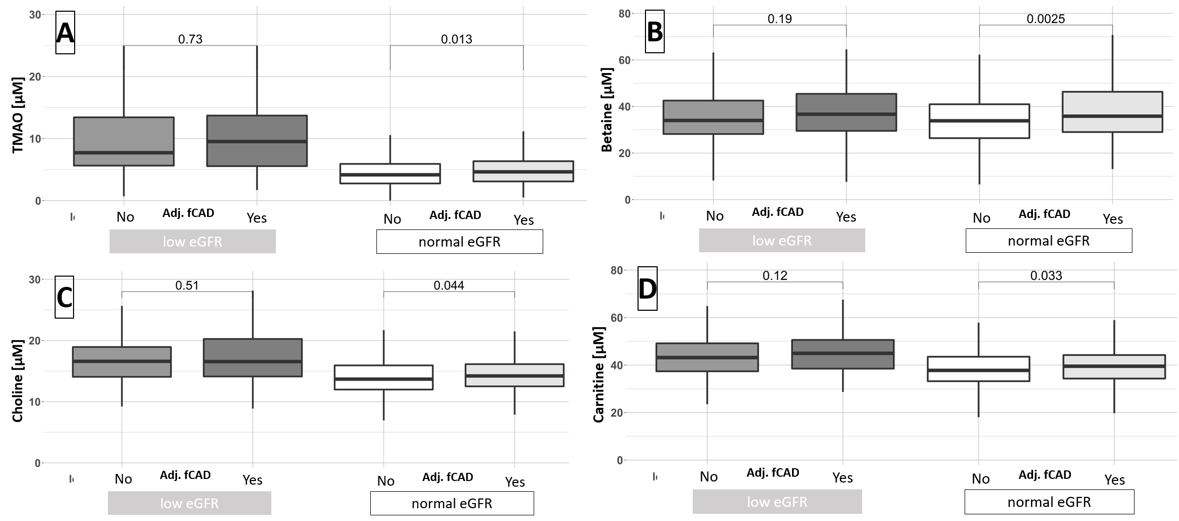


**Supplemental Fig. 2** – Subset analysis amongst patients with eGFR data (n = 919), comparing levels of TMAO, betaine, choline and carnitine between patients with and without fCAD.


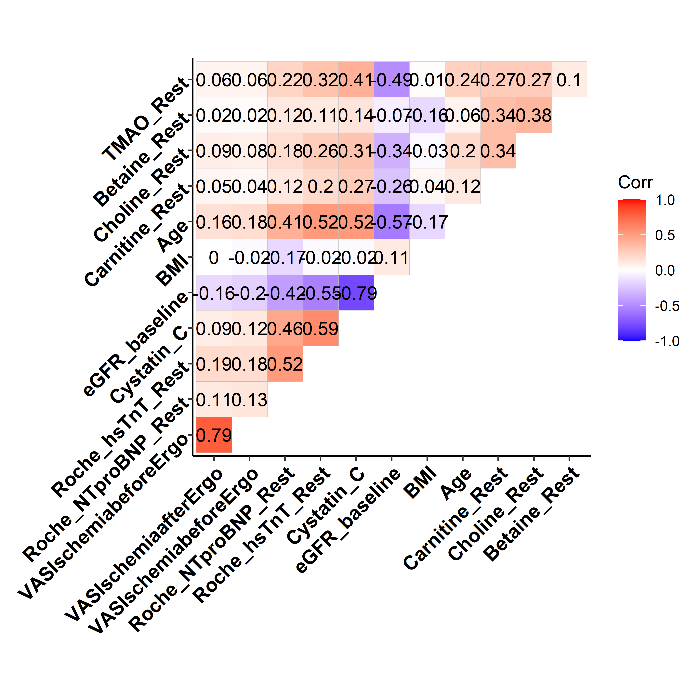


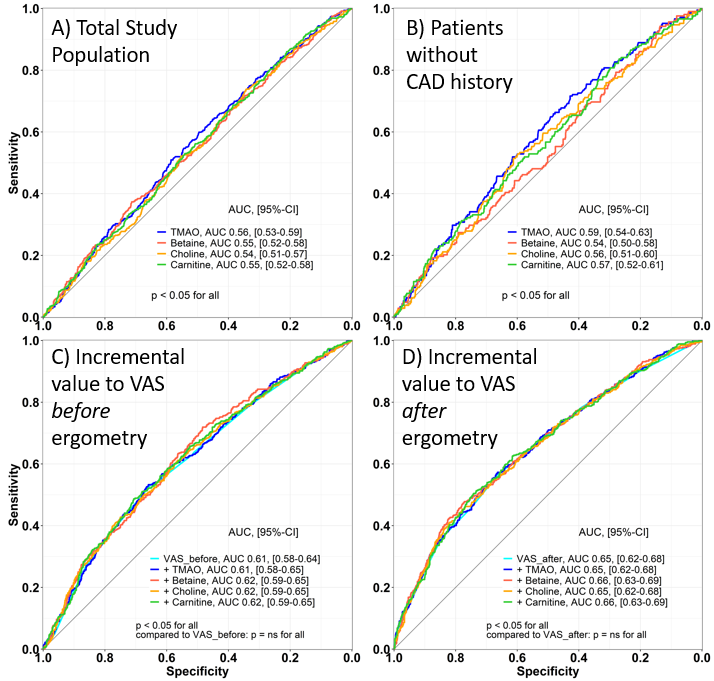
**Supplemental Fig. 3** – Correlation matrix containing Spearman’s Rho values as a measure of correlation between TMAO, the three precursors betaine, choline and carnitine as well as selected continuous variables.

**Supplemental Fig. 4** – ROC curves and AUC of TMAO and the precursors betaine, choline and carnitine among the total study population (A), among patients without CAD history (B), as incremental value to the clinical assessment by the treating physician (VAS) before ergometry (C) and as incremental value to VAS after ergometry (D).


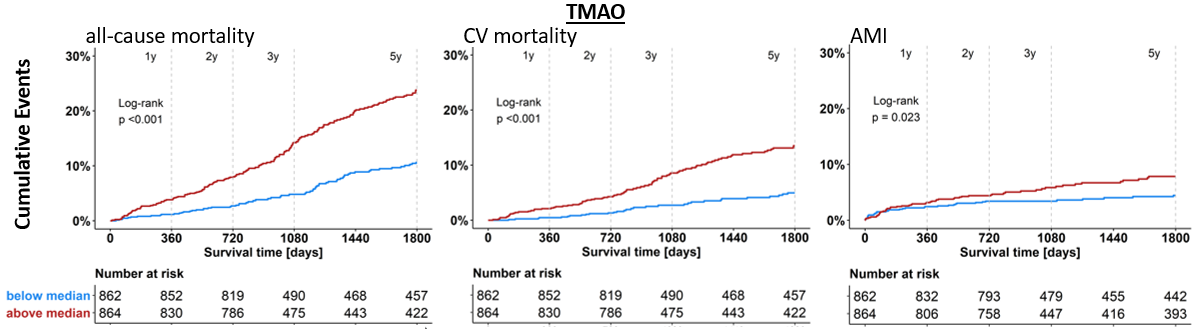

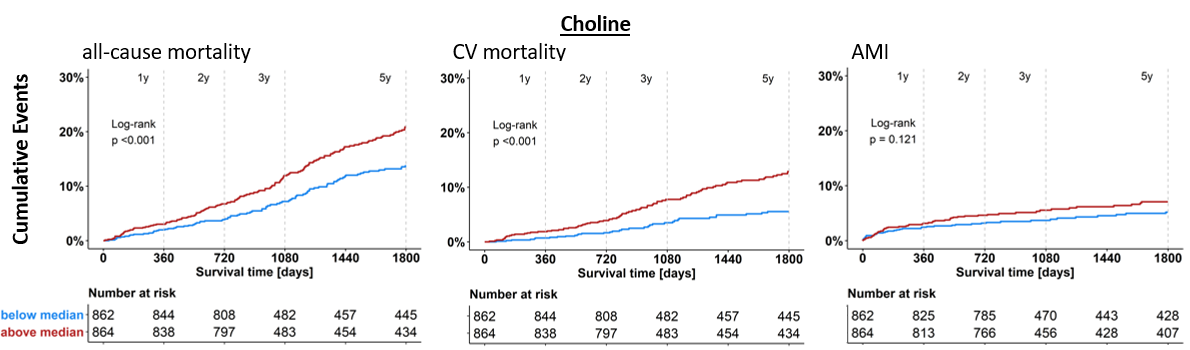

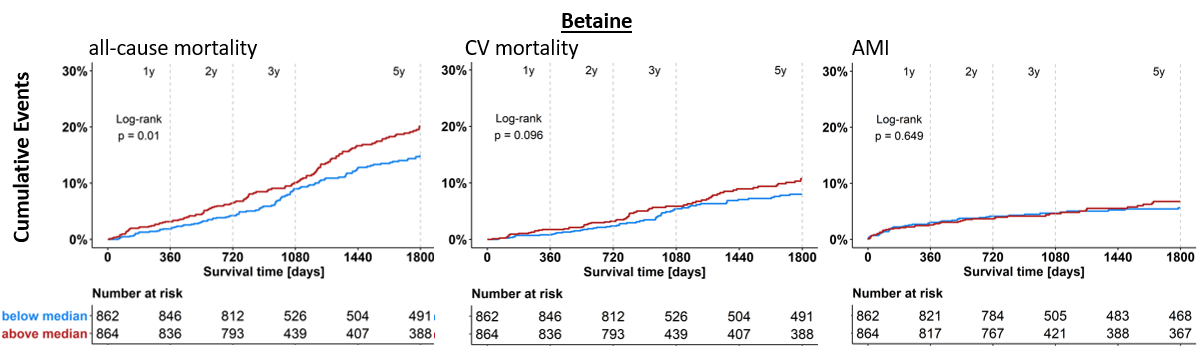

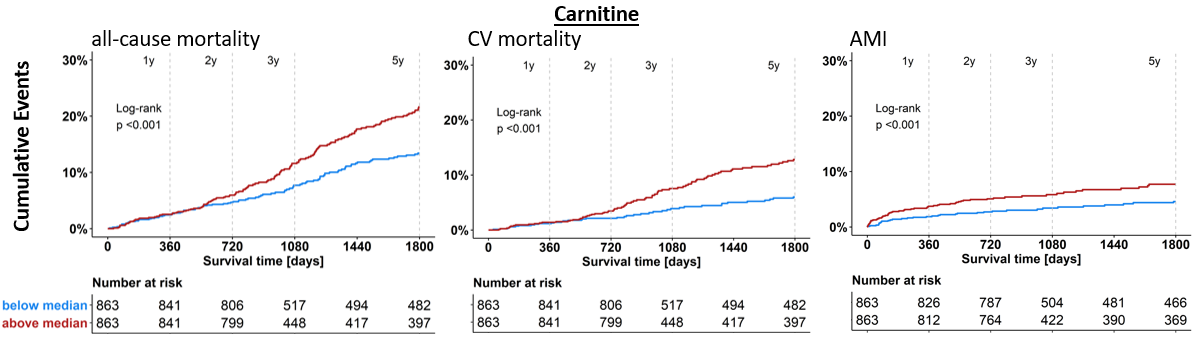


**Supplemental Fig. 5** **–** Kaplan Meier plots of TMAO and the precursors betaine, choline and carnitine stratified according to median levels of the markers for the endpoints all-cause mortality, cardiovascular mortality and acute myocardial infarction.


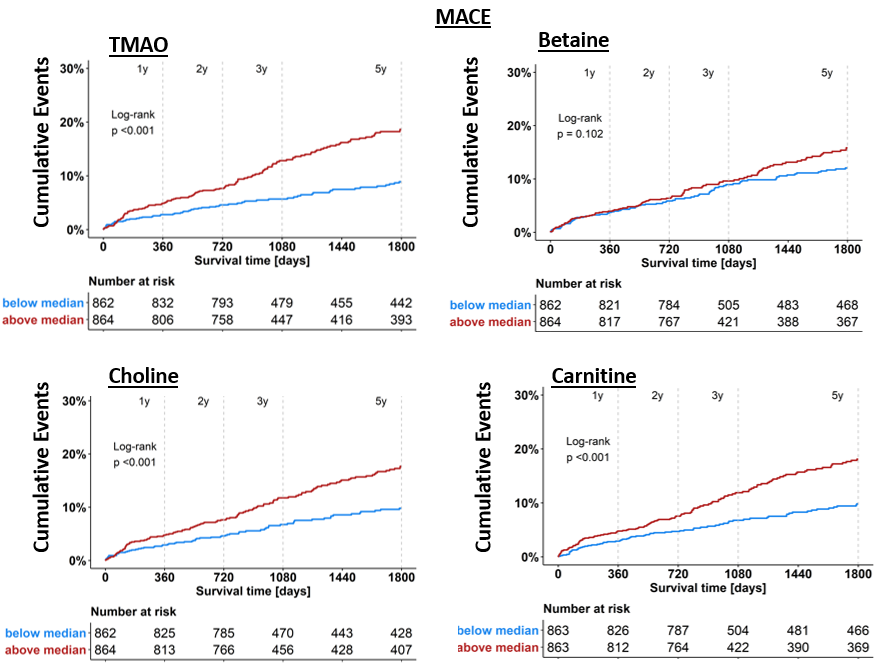


**Supplemental Fig. 6** **–** Kaplan Meier survival analysis of TMAO and the precursors betaine, choline and carnitine stratified according to median levels of the markers for the composite endpoint mace (cardiovascular death and AMI).

|  | **all-cause death** | |  | **CV-death** | |  | **AMI** | |  |
| --- | --- | --- | --- | --- | --- | --- | --- | --- | --- |
|  | **No** | **Yes** | **p-Value** | **Non-CV death** | **CV-death** | **p-Value** | **No** | **Yes** | **p-Value** |
| **N** | **1503** | **223** |  | **108** | **115** |  | **1638** | **88** |  |
| Age (years; median [IQR]) | 68.0 [59.0, 75.0] | 78.0 [71.0, 82.0] | <0.001 | 78.0 [71.0, 82.0] | 77.0 [71.0, 82.0] | 0.641 | 69.0 [60.0, 76.0] | 71.0 [63.0, 80.0] | 0.032 |
| Sex (Female (%)) | 979 (65.1) | 154 (69.1) | 0.258 | 77 (71.3) | 77 (67.0) | 0.562 | 1068 (65.2) | 65 (73.9) | 0.107 |
| BMI (median [IQR]) | 27.4 [24.6, 31.2] | 26.2 [23.5, 29.4] | <0.001 | 24.8 [23.0, 28.3] | 27.4 [24.0, 30.5] | 0.001 | 27.3 [24.4, 30.9] | 27.5 [24.0, 30.8] | 0.903 |
| Diabetes (%) | 367 (24.4) | 80 (35.9) | <0.001 | 34 (31.5) | 46 (40.0) | 0.210 | 411 (25.1) | 36 (40.9) | 0.002 |
| Ever Smoker (%) | 917 (61.0) | 153 (68.6) | 0.032 | 68 (63.0) | 85 (73.9) | 0.085 | 1020 (62.3) | 50 (56.8) | 0.312 |
| Family Hist. of CAD (%) | 448 (29.8) | 66 (29.6) | 1.000 | 28 (25.9) | 38 (33.0) | 0.304 | 481 (29.4) | 33 (37.5) | 0.119 |
| History of Hypertonia (%) | 1180 (78.5) | 195 (87.4) | 0.002 | 90 (83.3) | 105 (91.3) | 0.105 | 1296 (79.1) | 79 (89.8) | 0.014 |
| History of Hypercholesterolemia (%) | 1065 (70.9) | 164 (73.5) | 0.429 | 69 (63.9) | 95 (82.6) | 0.002 | 1157 (70.6) | 72 (81.8) | 0.029 |
| History of CAD (%) | 638 (42.4) | 126 (56.5) | <0.001 | 48 (44.4) | 78 (67.8) | <0.001 | 704 (43.0) | 60 (68.2) | <0.001 |
| History of MI (%) | 380 (25.3) | 70 (31.4) | 0.060 | 27 (25.0) | 43 (37.4) | 0.060 | 408 (24.9) | 42 (47.7) | <0.001 |
| History of PCI (%) | 510 (33.9) | 82 (36.8) | 0.407 | 31 (28.7) | 51 (44.3) | 0.018 | 543 (33.2) | 49 (55.7) | <0.001 |
| History of Bypass (%) | 186 (12.4) | 50 (22.4) | <0.001 | 15 (13.9) | 35 (30.4) | 0.004 | 217 (13.2) | 19 (21.6) | 0.037 |
| History of PAD (%) | 117 (7.8) | 49 (22.0) | <0.001 | 17 (15.7) | 32 (27.8) | 0.035 | 147 (9.0) | 19 (21.6) | 0.001 |
| History of Heart Failure (%) | 29 (1.9) | 25 (11.2) | <0.001 | 10 (9.3) | 15 (13.0) | 0.403 | 50 (3.1) | 4 (4.5) | 0.352 |
| Aortic Valve Disease (%) |  |  | <0.001 |  |  | 0.024 |  |  | 0.080 |
| None | 1319 (87.8) | 158 (70.9) |  | 79 (73.1) | 79 (68.7) |  | 1407 (85.9) | 70 (79.5) |  |
| Stenosis | 70 (4.7) | 27 (12.1) |  | 7 (6.5) | 20 (17.4) |  | 91 (5.6) | 6 (6.8) |  |
| Insufficiency | 112 (7.5) | 37 (16.6) |  | 22 (20.4) | 15 (13.0) |  | 138 (8.4) | 11 (12.5) |  |
| Combined | 2 (0.1) | 1 (0.4) |  | 0 (0.0) | 1 (0.9) |  | 2 (0.1) | 1 (1.1) |  |
| Mitral Valve Disease (%) |  |  | <0.001 |  |  | 0.041 |  |  | 0.047 |
| None | 1207 (80.3) | 129 (58.1) |  | 70 (65.4) | 59 (51.3) |  | 1277 (78.0) | 59 (67.0) |  |
| Stenosis | 1 (0.1) | 1 (0.5) |  | 0 (0.0) | 1 (0.9) |  | 2 (0.1) | 0 (0.0) |  |
| Insufficiency | 295 (19.6) | 92 (41.4) |  | 37 (34.6) | 55 (47.8) |  | 358 (21.9) | 29 (33.0) |  |
| History of Stoke or TIA (%) | 107 (7.1) | 34 (15.2) | <0.001 | 13 (12.0) | 21 (18.3) | 0.263 | 136 (8.3) | 5 (5.7) | 0.547 |
| History of COPD (%) | 109 (7.3) | 50 (22.4) | <0.001 | 22 (20.4) | 28 (24.3) | 0.523 | 150 (9.2) | 9 (10.2) | 0.705 |
| Aspirin (%) | 885 (58.9) | 130 (58.3) | 0.884 | 57 (52.8) | 73 (63.5) | 0.135 | 941 (57.4) | 74 (84.1) | <0.001 |
| Thienopyridine (%) | 97 (6.5) | 15 (6.7) | 0.884 | 5 (4.6) | 10 (8.7) | 0.289 | 104 (6.3) | 8 (9.1) | 0.271 |
| Nitroglycerine (%) | 107 (7.1) | 40 (17.9) | <0.001 | 15 (13.9) | 25 (21.7) | 0.162 | 131 (8.0) | 16 (18.2) | 0.003 |
| Beta-Blocker (%) | 767 (51.0) | 139 (62.3) | 0.002 | 60 (55.6) | 79 (68.7) | 0.053 | 852 (52.0) | 54 (61.4) | 0.100 |
| Calcium-Antagonist (%) | 335 (22.3) | 52 (23.3) | 0.731 | 21 (19.4) | 31 (27.0) | 0.207 | 358 (21.9) | 29 (33.0) | 0.018 |
| Amiadarone (%) | 34 (2.3) | 8 (3.6) | 0.241 | 5 (4.6) | 3 (2.6) | 0.488 | 40 (2.4) | 2 (2.3) | 1.000 |
| Diuretic (%) | 565 (37.6) | 141 (63.2) | <0.001 | 60 (55.6) | 81 (70.4) | 0.026 | 661 (40.4) | 45 (51.1) | 0.058 |
| ACE-Inhibitor (%) | 430 (28.6) | 89 (39.9) | 0.001 | 42 (38.9) | 47 (40.9) | 0.786 | 485 (29.6) | 34 (38.6) | 0.075 |
| AR-Blocker (%) | 504 (33.5) | 72 (32.3) | 0.761 | 34 (31.5) | 38 (33.0) | 0.886 | 545 (33.3) | 31 (35.2) | 0.728 |
| Statin (%) | 860 (57.2) | 133 (59.6) | 0.514 | 53 (49.1) | 80 (69.6) | 0.003 | 935 (57.1) | 58 (65.9) | 0.121 |
| Phenprocoumon (%) | 141 (9.4) | 60 (26.9) | <0.001 | 26 (24.1) | 34 (29.6) | 0.369 | 189 (11.5) | 12 (13.6) | 0.498 |
| PPI (%) | 450 (29.9) | 93 (41.7) | 0.001 | 44 (40.7) | 49 (42.6) | 0.788 | 504 (30.8) | 39 (44.3) | 0.009 |
| VAS before Ergo (median [IQR]) | 40.0 [20.0, 60.0] | 50.0 [30.0, 60.0] | <0.001 | 50.0 [30.0, 60.0] | 50.0 [30.0, 70.0] | 0.754 | 40.0 [20.0, 60.0] | 50.0 [30.0, 70.0] | <0.001 |
| VAS after Ergo (median [IQR]) | 30.0 [20.0, 60.0] | 50.0 [30.0, 70.0] | <0.001 | 45.0 [30.0, 60.0] | 50.0 [30.0, 70.0] | 0.752 | 35.0 [20.0, 60.0] | 60.0 [30.0, 80.0] | <0.001 |
| Echo_LVEF (median [IQR]) | 59.0 [52.5, 62.5] | 52.5 [41.0, 60.0] | <0.001 | 55.0 [43.8, 60.0] | 50.0 [40.2, 60.0] | 0.482 | 58.0 [50.0, 62.0] | 55.0 [45.0, 60.0] | 0.005 |
| eGFR_baseline (median [IQR]) | 82.4 [66.2, 93.8] | 55.0 [37.4, 81.3] | <0.001 | 63.3 [39.4, 84.4] | 51.1 [33.8, 76.5] | 0.030 | 80.3 [61.1, 92.5] | 69.6 [39.2, 88.5] | 0.005 |
| Cystatin-C [RFU/1000] | 2.4 [2.1, 2.8] | 3.4 [2.7, 4.2] | <0.001 | 3.2 [2.5, 4.0] | 3.6 [3.0, 4.8] | 0.001 | 2.5 [2.1, 3.0] | 2.7 [2.3, 3.9] | 0.003 |
| TMAO (median [IQR]) | 4.6 [3.1, 7.0] | 6.5 [4.5, 12.0] | <0.001 | 6.0 [4.0, 10.1] | 7.0 [4.8, 12.8] | 0.041 | 4.8 [3.1, 7.4] | 5.8 [4.0, 10.6] | 0.003 |
| Betaine (median [IQR]) | 34.4 [28.2, 42.6] | 35.9 [28.8, 46.6] | 0.032 | 35.9 [28.0, 44.4] | 37.0 [30.1, 47.5] | 0.346 | 34.6 [28.2, 43.2] | 35.0 [29.5, 41.8] | 0.677 |
| Choline (median [IQR]) | 14.2 [12.3, 16.6] | 15.6 [13.0, 19.2] | <0.001 | 14.7 [12.7, 19.0] | 16.1 [14.1, 19.2] | 0.025 | 14.3 [12.4, 16.8] | 15.3 [12.7, 18.4] | 0.014 |
| Carnitine (median [IQR]) | 39.2 [34.2, 44.4] | 41.4 [36.7, 48.8] | <0.001 | 39.9 [34.9, 45.8] | 44.1 [38.4, 51.1] | 0.002 | 39.3 [34.3, 44.7] | 42.5 [37.0, 48.9] | 0.002 |

**Supplemental Table 1** – Patient Baseline Characteristics stratified by the outcome events all-cause death, cardiovascular death and AMI. Patient Baseline Characteristics stratified according the outcome events all-cause death, cardiovascular death and AMI (ACE inhibitor - angiotensin-converting-enzyme inhibitor; AMI - acute myocardial infarction; ARB - Angiotensin II Receptor Blockers; BMI - body mass index; CAD - coronary artery disease; CABG - Coronary artery bypass grafting; COPD - chronic obstructive pulmonary disease; PAD – peripheral artery disease; PCI - Percutaneous coronary intervention; TIA - transient ischemic attack; VAS - clinical assessment of cardiologist for presence of functionally relevant CAD before and after cardiac stress testing but prior to imaging); PPI – proton pump inhibitor

|  | **Overall** | **fCAD** | | |  |
| --- | --- | --- | --- | --- | --- |
|  | **N = 689** | **No N=453** | **Yes N=236** | | **p-Value** |
| Age (yrs; median [IQR]) | 67.0 [58.0, 75.0] | 66.0 [56.0, 74.0] | 69.0 [60.8, 75.0] | | 0.010 |
| Sex (Male (%)) | 465 (67.5) | 272 (60.0) | 193 (81.8) | | <0.001 |
| BMI (median [IQR]) | 27.4 [24.3, 31.5] | 27.6 [24.1, 31.7] | 27.3 [24.6, 31.0] | | 0.513 |
| ***Medical History:*** |  |  |  | |  |
| Diabetes (%) | 176 (25.5) | 102 (22.5) | 74 (31.4) | | 0.013 |
| Ever Smoker (%) | 455 (66.0) | 295 (65.1) | 160 (67.8) | | 0.499 |
| Family History of CAD (%) | 192 (27.9) | 127 (28.0) | 65 (27.5) | | 0.929 |
| History of Hypertension (%) | 535 (77.6) | 339 (74.8) | 196 (83.1) | | 0.016 |
| History Hypercholesterolemia (%) | 470 (68.2) | 290 (64.0) | 180 (76.3) | | 0.001 |
| History of CAD (%) | 288 (41.8) | 164 (36.2) | 124 (52.5) | | <0.001 |
| History of MI (%) | 182 (26.4) | 99 (21.9) | 83 (35.2) | | <0.001 |
| History of PCI (%) | 231 (33.5) | 129 (28.5) | 102 (43.2) | | <0.001 |
| History of Bypass (%) | 77 (11.2) | 33 (7.3) | 44 (18.6) | | <0.001 |
| History of PAD (%) | 62 (9.0) | 39 (8.6) | 23 (9.7) | | 0.674 |
| History of Heart Failure (%) | 17 (2.5) | 12 (2.6) | 5 (2.1) | | 0.799 |
| Aortic Valve Disease (%) |  |  |  | | 0.163 |
| None | 599 (86.9) | 400 (88.3) | 199 (84.3) | |  |
| Stenosis | 32 (4.6) | 17 (3.8) | 15 (6.4) | |  |
| Insufficiency | 57 (8.3) | 36 (7.9) | 21 (8.9) | |  |
| Combined | 1 (0.1) | 0 (0.0) | 1 (0.4) | |  |
| Mitral Valve Disease (%) |  |  |  | | 0.003 |
| None | 535 (77.6) | 368 (81.2) | 167 (70.8) | |  |
| Stenosis | 1 (0.1) | 1 (0.2) | 0 (0.0) | |  |
| Insufficiency | 153 (22.2) | 84 (18.5) | 69 (29.2) | |  |
| History of Stoke or TIA (%) | 56 (8.1) | 35 (7.7) | 21 (8.9) | | 0.660 |
| History of COPD (%) | 68 (9.9) | 46 (10.2) | 22 (9.3) | | 0.789 |
| ***Baseline Medication:*** |  |  |  | |  |
| Aspirin (%) | 405 (58.8) | 244 (53.9) | 161 (68.2) | | <0.001 |
| Thienopyridine (%) | 56 (8.1) | 29 (6.4) | 27 (11.4) | 0.027 | |
| Nitroglycerine (%) | 52 (7.5) | 22 (4.9) | 30 (12.7) | | <0.001 |
| Beta-Blocker (%) | 350 (50.8) | 200 (44.2) | 150 (63.6) | | <0.001 |
| Calcium-Antagonist (%) | 154 (22.4) | 109 (24.1) | 45 (19.1) | | 0.149 |
| Amiadarone (%) | 14 (2.0) | 7 (1.5) | 7 (3.0) | | 0.256 |
| Diuretic (%) | 238 (34.5) | 151 (33.3) | 87 (36.9) | | 0.355 |
| ACE-Inhibitor (%) | 221 (32.1) | 131 (28.9) | 90 (38.1) | | 0.016 |
| AR-Blocker (%) | 194 (28.2) | 124 (27.4) | 70 (29.7) | | 0.533 |
| Statin (%) | 382 (55.4) | 222 (49.0) | 160 (67.8) | | <0.001 |
| Phemprocoumon (%) | 75 (10.9) | 40 (8.8) | 35 (14.8) | | 0.020 |
| Protonepump Inhibitor (%) | 223 (32.4) | 157 (34.7) | 66 (28.0) | | 0.086 |
| VAS before Ergo (median [IQR]) | 59.0 [52.4, 63.0] | 30.0 [20.0, 50.0] | 50.0 [30.0, 70.0] | | <0.001 |
| VAS after Ergo (median [IQR]) | 86.4 [76.0, 95.5] | 30.0 [20.0, 50.0] | 50.0 [23.8, 80.0] | | <0.001 |
| Echo_LVEF (median [IQR]) | 2.4 [2.1, 2.7] | 60.0 [55.0, 63.5] | 55.0 [46.0, 62.0] | | 0.001 |
| eGFR_baseline (median [IQR]) | 4.3 [2.9, 6.1] | 87.5 [77.8, 98.0] | 84.7 [74.2, 92.7] | | 0.003 |
| Cystatin_C [RFU/1000] | 2.4 [2.1, 2.7] | 2.3 [2.1, 2.7] | 2.5 [2.1, 2.7] | | 0.012 |
| TMAO (median [IQR]) | 4.3 [2.9, 6.1] | 4.2 [2.8, 5.9] | 4.6 [3.1, 6.3] | | 0.013 |
| Betaine (median [IQR]) | 34.4 [28.2, 42.3] | 33.9 [26.4, 41.0] | 35.8 [29.0, 46.3] | | 0.003 |
| Choline (median [IQR]) | 13.8 [12.2, 16.0] | 13.7 [12.0, 15.9] | 14.2 [12.5, 16.1] | | 0.044 |
| Carnitine (median [IQR]) | 38.2 [33.7, 43.9] | 37.8 [33.2, 43.5] | 39.5 [34.3, 44.2] | | 0.033 |

**Supplemental Table 2** – Subgroup Patient Baseline Characteristics in Patients with normal eGFR (ACE inhibitor - angiotensin-converting-enzyme inhibitor; AMI - acute myocardial infarction; ARB - Angiotensin II Receptor Blockers; BMI - body mass index; CAD - coronary artery disease; CABG - Coronary artery bypass grafting; COPD - chronic obstructive pulmonary disease; PAD – peripheral artery disease; PCI - Percutaneous coronary intervention; TIA - transient ischemic attack; VAS - clinical assessment of cardiologist for presence of functionally relevant CAD before and after cardiac stress testing (ergometry) but prior to imaging, eGFR at baseline and Cystatin-C in relative fluorescent unit (RFU)/1000).

|  | | **TMAO** | **Betaine** | **Choline** | **Carnitine** |
| --- | --- | --- | --- | --- | --- |
| **Univariate** | OR (95%CI),  p-Value | *1.19**  *(1.08, 1.31),*  *p < 0.001* | *1.43**  *(1.15, 1.77),*  *p = 0.001* | *1.49**  *(1.09, 2.03), p = 0.011* | *1.71**  *(1.23, 2.37),*  *p = 0.001* |
| **Model 1** | OR (95%CI),  p-Value | *1.12**  *(1.01, 1.24), p = 0.035* | 1.06  (0.85, 1.33), p = 0.607 | 1.02  (0.73, 1.42), p = 0.897 | 1.21  (0.86, 1.71),  p = 0.273 |
| **Model 1 + Cystatin-C** | OR (95%CI),  p-Value | 1.08 (0.96,1.2),  p = 0.196 | 1.06 (0.84,1.33),  p = 0.631 | 0.90  (0.64,1.28),  p = 0.566 | 1.11 (0.78,1.57),  p = 0.576 |
| **Model 2** | OR (95%CI),  p-Value | 1.07  (0.96,1.19),  p = 0.240 | 1.12 (0.88,1.42),  p = 0.347 | 0.91 (0.65,1.27),  p =0.578 | 1.12 (0.78,1.59),  p = 0.537 |
| **Subset (n = 689): subset of patients with normal eGFR data** | | | | | |
| **Univariate** | OR (95%CI),  p-Value | *1.25**  *(1.06,1.46),*  *p = 0.006* | *1.80**  *(1.3,2.5),*  *p < 0.001* | 1.59  (0.98,2.57),  p = 0.061 | *1.71**  *(1.01,2.88),*  *p = 0.045* |
| **Model 1** | OR (95%CI),  p-Value | 1.17 (0.99,1.38),  p = 0.064 | 1.28 (0.9,1.82),  p = 0.174 | 1.19 (0.72,1.98),  p = 0.502 | 1.22 (0.71,2.1),  p = 0.472 |

**Supplemental Table 3** **–** Odds Ratio (OR) with 95%-confidence interval from logistic regression models. Model 1 adjustment: age, sex, history of CAD; Model 1 + Cystatin-C; Model 2 adjustment: pre-defined patient characteristics, cardiovascular risk factors and medical history including age, sex, CAD history, history of smoking/ + diabetes/+ AMI/ +heart failure, history of stroke/TIA, positive cardiovascular family history. Markers were log2-transformed. Subset analysis was performed on patients with normal renal function (eGFR >= 60).

|  | **Outcome** | **Univariate** | **Model 1** | **Model 1 + Cystatin-C** | **Model 2** | **Model 2 + Cystatin-C** |
| --- | --- | --- | --- | --- | --- | --- |
| **TMAO HR**  **(95%-CI), p-Value** | **all-cause death** | *2.51* (1.88, 3.35),*  *p < 0.001* | *1.78* (1.32, 2.38),*  *p < 0.001* | *1.67* (1.24, 2.25),*  *p < 0.001* | *1.67* (1.24, 2.26),*  *p < 0.001* | *1.58* (1.16, 2.14),*  *p = 0.003* |
|  | **CV death** | *2.92* (1.92, 4.42),*  *p <0.001* | *2.06* (1.35, 3.14),*  *p < 0.001* | *1.97* (1.28, 3.04),*  *p = 0.002* | *1.79* (1.16, 2.76),*  *p = 0.008* | *1.66* (1.07, 2.59),*  *p = 0.025* |
|  | **AMI** | *1.63* (1.06, 2.47),*  *p = 0.025* | 1.40 (0.89, 2.16),  p = 0.137 |  |  |  |
| **Betaine**  **HR**  **(95%-CI), p-Value** | **all-cause death** | *1.41* (1.08, 1.84),*  *p = 0.011* | 1.18 (0.89, 1.54),  p = 0.241 |  |  |  |
|  | **CV death** | 1.36 (0.94, 1.97),  p = 0.098 |  |  |  |  |
|  | **AMI** | 1.21 (0.80, 1.82),  p = 0.369 |  |  |  |  |
| **Choline**  **HR**  **(95%-CI), p-Value** | **all-cause death** | *1.60* (1.22, 2.09),*  *p < 0.001* | 1.14 (0.87, 1.50),  p = 0.344 |  |  |  |
|  | **CV death** | *2.39* (1.60, 3.56),*  *p < 0.001* | *1.76* (1.17, 2.65),*  *p = 0.006* | *1.79* (1.18, 2.72),*  *p = 0.006* | *1.53* (1.01, 2.33),*  *p = 0.047* | 1.51 (0.98, 2.33)  p = 0.059 |
|  | **AMI** | 1.40 (0.91, 2.13),  p = 0.123 |  |  |  |  |
| **Carnitine**  **HR**  **(95%-CI), p-Value** | **all-cause death** | *1.61* (1.23, 2.11),*  *p < 0.001* | *1.31* (1.00, 1.72),*  *p = 0.049* | 1.10 (0.83, 1.45)  p = 0.481 | 1.23 (0.94, 1.63),  p = 0.132 |  |
|  | **CV death** | *2.12* (1.44, 3.12),*  *p = 0.001* | *1.69* (1.14, 2.49),*  *p = 0.008* | 1.36 (0.91, 2.04),  p = 0.129 | *1.53* (1.03, 2.28),*  *p = 0.036* | 1.24 (0.82, 1.87),  p = 0.302 |
|  | **AMI** | *1.81* (1.18, 2.76),*  *p = 0.006* | *1.54* (1.01, 2.37),*  *p = 0.046* | 1.39 (0.90, 2.14),  P = 0.142 | *1.61 * (1.04, 2.48),*  *p = 0.030* | 1.41 (0.91, 2.19),  p = 0.129 |

**Supplemental Table 4** – Hazard Ratios (HR) of the cox Regression model comparing marker levels of TMAO, betaine, choline and carnitine, comparing above the median to below the median, with 95%-CI with p-values. Model 1, adjusted for age, gender and CAD history and model 1 applied to patients without known CAD history. ; Model 2 was adjusted for pre-defined patient characteristics, cardiovascular risk factors and medical history including age, gender, body mass index, smoking history, positive cardiovascular family history, hypertension, hypercholesterolemia, history of diabetes, history of stroke/TIA, history of coronary artery disease, previous acute myocardial infarction, history of heart failure and adjudicated functionally relevant coronary artery disease.
